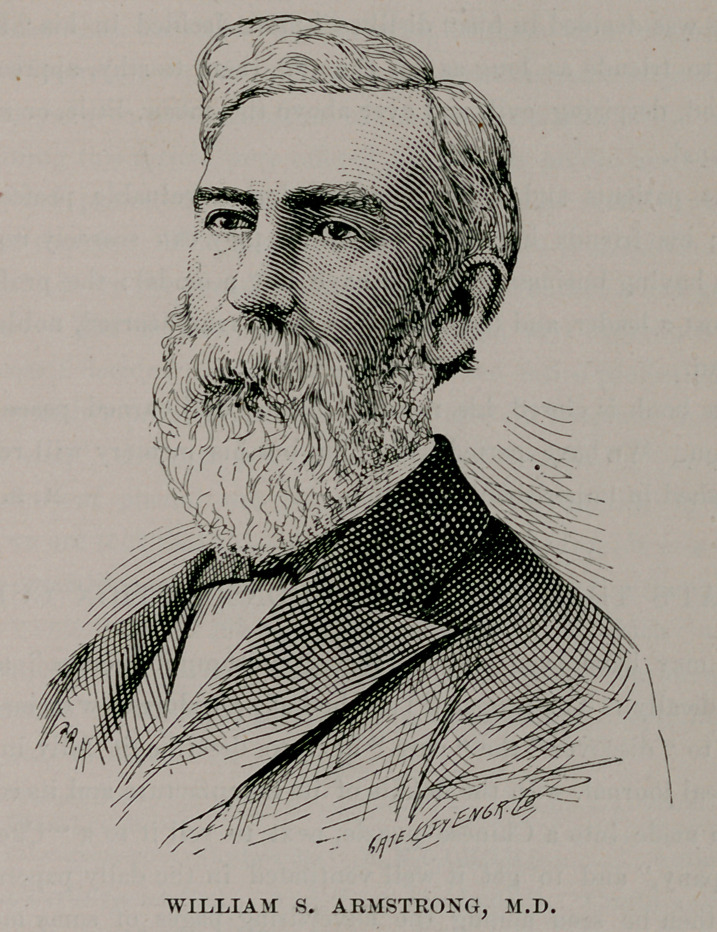# Dr. William S. Armstrong

**Published:** 1896-03

**Authors:** 


### DR. WILLIAM S. ARMSTRONG.

On the evening of February 11, 1896, Dr. Wm. S. Armstrong died unexpectedly at the Grady Hospital, just as he was preparing to call to order the Medical Board, of which he was president. Dr. Armstrong was Professor of Anatomy and Clinical Surgery in the Atlanta Medical College, and its Treasurer; President of the Grady Hospital Board; President of the State Anatomical Board, and a member of various medical societies.

If it be granted to those who have passed away to look back upon their lives and into the hearts of those whom they have left behind, he realizes now the good which he did, the reputation which he won, and knows the love which exists in the hearts of his associates here. He had desired to be spared a long and lingering illness, worse than death to one of his energy, but had hoped that the end might come as a sudden summons to leave his labors.

In the institution which he loved well, in the midst of work which was his delight, his face lost its consciousness of earthly things, his ear caught the mandate to cease his labor, and the weary heart gratefully yielded to the call to rest.

It was fitting that he should die at his post, it was merciful that his family was spared the shock of witnessing his sudden taking away, and the manner and time of his death riveted the bonds of his colleagues' affection.

The writer first got to know Dr. Armstrong a little over ten years ago. Work had to be well done, the doing of which was not always practicable; faults were plain, but there was never a word of impatience, never a lapse of courtesy, never a forgetfulness of kindness on his part. At times hasty in his speech, severe in his judgment, and bitter in his condemnation of wrong, he never forgot his honorable character of gentleman. Though of a nervous temperament, with exhaustless energy, his life was well balanced and systematized. He had, as a young man, so many hours for work, so many for study, and so many for rest and social enjoyment.

WILLIAM S. ARMSTRONG, M.D.

Prompt in business, in fact the model of an accurate, perfect business man, prompt at his lectures, prompt in answer to the closing bell—always prompt, no one's time lost through him, his own saved by him.

Long enough with his college to have become a routine member following his work as a treadmill, he did not. To the final moment he was active, progressive, and enthusiastic in his college duties.

Nervous in action and speedy as an operator, few surgeons anywhere can boast of greater success.

He was decided in his "dislikes," more decided in his "likes," true to friends as long as he thought them worthy, appreciative of good, despising evil, and ever above the mean, little, or unprofessional.

His patients and institutions have lost a valuable professional man; his friends have lost one whom they can scarcely replace; those having business with him have lost a model; the profession has lost a leader, and the world has lost a true-hearted, noble gentleman.

His book is closed, his record is made, well-earned peace is his portion. We have parted with him, but his memory will remain, cherished in imperishable affection. M. B. H.

## Figures and Tables

**Figure f1:**